# Changes in Estrogen Receptor ERβ (ESR2) Expression without Changes in the Estradiol Levels in the Prostate of Aging Rats

**DOI:** 10.1371/journal.pone.0131901

**Published:** 2015-07-06

**Authors:** Mônica Morais-Santos, Aryane E. B. Nunes, André G. Oliveira, Júnia Dayrell Moura-Cordeiro, Germán A. B. Mahecha, Maria Christina W. Avellar, Cleida A. Oliveira

**Affiliations:** 1 Department of Morphology, Institute of Biological Sciences, Universidade Federal de Minas Gerais, Belo Horizonte, Minas Gerais, Brazil; 2 Department of Pharmacology, Section of Experimental Endocrinology, Escola Paulista de Medicina, Universidade Federal de São Paulo, São Paulo, São Paulo, Brazil; Louisiana State University Health Sciences center, UNITED STATES

## Abstract

Although the prostate is androgen-dependent, it is also influenced by estrogens, which act via the estrogen receptors ERα and ERβ. In the prostate, ERβ is highly expressed in the epithelium and appears to participate in the regulation of cell proliferation, apoptosis and differentiation. Evidence shows that ERβ is decreased in malignant prostate, suggesting that it plays an important role in protecting this tissue. Despite the relationship between reductions in ERβ and abnormal growth of the gland, little is known about the age-dependent variation of this receptor. Therefore, we aimed to investigate ERβ expression in the prostatic lobes of aging Wistar rats (3 to 24 months). Histopathological alterations, including hyperplasia, intraluminal concretions, nuclear atypia and prostate intraepithelial neoplasias (PIN), were observed in the prostates of aging rats. Epithelial proliferation led to cribriform architecture in some acini, especially in the ventral prostate (VP). In the VP, areas of epithelial atrophy were also observed. Furthermore, in the lateral prostate, there was frequent prostatitis. Immunohistochemistry revealed that the expression of ERβ is reduced in specific areas related to PIN, atrophic abnormalities and cellular atypia in the prostate epithelium of senile rats. Corroborating the involvement of the receptor with proliferative activity, the punctual reduction in ERβ paralleled the increase in cell proliferation especially in areas of PIN and nuclear atypies. The decrease in ERβ reactivity occurred in a hormonal milieu characterized by a constant concentration of estradiol and decreased plasmatic and tissue DHT. This paper is a pioneering study that reveals focal ERβ reduction in the prostate of aging rats and indicates a potential disorder in the ERβ pathway. These data corroborate previous data from humans and dogs that silencing of this receptor may be associated with premalignant or malignant conditions in the prostate.

## Introduction

Although the prostate is a classic androgen-dependent organ, evidence indicates that the gland is also influenced by estrogens [1, 2], which can act via the estrogen receptors ERα (ERS1) and ERβ (ERS2) [3]. The expression of these receptors varies in intensity and distribution in rat and human prostate. In both species, ERα is expressed in few cells of the stroma, whereas ERβ is highly expressed in the epithelium and in some stromal cells [4–7].

ERβ has been implicated in several biological functions in the prostate, including the regulation of cell proliferation, apoptosis and cell differentiation [8–15]. Interestingly, evidence suggests that ERβ expression is highly decreased in malignant prostate tissue, reaching nearly undetectable levels with tumor progression [6, 16–23]. Most impressively, the reintroduction of ERβ triggers apoptosis and decreases the proliferation and invasiveness of malignant cells [10]. In addition, prostate hyperplasia has been described in mice lacking ERβ (βERKO) [8, 24]. Together, these findings suggest that ERβ plays a role in protecting the prostate against abnormal growth.

Although the origin of prostate cancer is still not completely understood, aging is the main risk factor for men, considering that both the incidence and mortality increase exponentially after 50 years of age [25]. This fact may be related to hormonal changes associated with aging, particularly the increase in the estrogen:androgen ratio [26–30]. Despite the evidence that ERβ plays an important role in prostate physiology and the close relationship between the reduction of this receptor and abnormal growth of the aging gland, little is known about the occurrence of age-dependent variation in epithelial ERβ expression. Therefore, to gain insight into this relevant issue, we aimed to investigate the pattern of ERβ expression in the prostatic lobes of aging adult rats.

## Materials and Methods

### Animals

The study was performed in adult male Wistar rats at 3, 6, 12, 18 and 24 months of age. The rats were housed in the animal facility at the Instituto de Ciências Biológicas of the Universidade Federal de Minas Gerais, Brazil. The animals were maintained under a constant light cycle (12 h of light and 12 h of darkness) and temperature (22°C) and received pelleted chow (Nuvital Nutrientes S.A, Colombo, Brazil) and water *ad libitum*. The beginning of the investigation was determined because the expression of ERβ mRNA reaches the adult pattern levels at 90 days of age [31].

### Ethics Statement

The experimental procedures were approved by the Ethical Committee for Animal Experimentation of the Universidade Federal de Minas Gerais (CETEA/UFMG—process 286/2008).

### Tissue preparation

The rats were weighed, anesthetized (i.p. 100 mg/kg sodium thiopental) and transcardially perfused with Ringer’s solution, followed by 10% neutral buffered formalin (NBF). After fixation, the prostatic complex, comprised of the ventral, dorsal, lateral and anterior lobes, was dissected and weighed, and the relative organ weights were calculated per 100 g of body weight (BW). The glands were sectioned and processed for paraffin (Histosec Pastilles, Merck, Darmstadt, Germany) or glycolmethacrylate embedding (Technovit 7100, Heraeus Kulzer, Wehrheim, Germany). Alternatively, animals were perfused with Ringer’s solution, and the freshly dissected lobes were weighed, immediately frozen in liquid nitrogen and kept at -80°C until use.

### Histopathology

To evaluate possible morphological and/or pathological alterations in the prostate lobes, fragments of each lobe in glycolmethacrylate were sectioned (3 μm) and stained with hematoxylin and eosin (H&E) or periodic acid-Schiff (PAS) followed by Mayer’s hematoxylin counterstaining. The pathological descriptions were based on The Consensus Report from the Bar Harbor Meeting of the Mouse Models of Human Cancer Consortium Prostate Pathology Committee [32].

### Immunohistochemistry

Tissue sections from prostatic lobes of rats at different ages (n = 5 per age) were performed in parallel, and the staining for ERβ detection was performed in triplicate to confirm the results. Additionally, staining for high molecular weight cytokeratin (CK HMW) was performed in the ventral and dorsal prostates to recognize basal cell distribution. Cell proliferation was also investigated in the ventral prostate by using Minichromosome Maintenance 7 (MCM7) as markers. NBF-fixed tissues were embedded in paraffin, and the obtained sections (5 μm) were subjected to microwave antigen retrieval followed by blocking of endogenous biotin and avidin using a commercial kit (Avidin/Biotin blocking kit, Vector Laboratories, Burlingame, USA). The sections were incubated with 10% normal goat serum to block non-specific antibody binding, prior to incubation with primary antibodies. For ERβ detection, the sections were incubated with mouse monoclonal anti-human ERβ (NCL-ERβ, Leica Biosystems, Wetzlar, Germany) diluted 1:25 in Tris-HCl buffer solution (TBS) at 4°C for approximately 40 hours or with mouse monoclonal anti-human CK HMW (34βE12, Dako, Glostrup, DK) diluted 1:200 in phosphate buffer solution (PBS) at 4°C overnight. For detection of cell proliferation, the sections were incubated with mouse monoclonal anti-MCM7 (Thermo Scientific, Waltham, USA), diluted 1:300 in PBS, at 4°C, overnight. The negative controls were performed in the absence of primary antibodies. After washing in saline buffer, the sections were exposed to a biotinylated secondary goat anti-mouse antibody diluted 1:50 for ERβ and 1:100 for MCM7 and CK HMW for 1 h. After this step, the sections were incubated for 45 min with streptavidin (Horseradish Peroxidase Streptavidin, Vector Laboratories, Burlingame, USA) for the ERβ assay and with avidin-biotin conjugated with peroxidase (Vectastain Elite ABC kit, Vector Laboratories—Burlingame, USA) for CK HMW. The immunoreactions for both antibodies were visualized using 0.55 mM diaminobenzidine and 0.01% H_2_O_2_ in 0.05 M Tris-HCl buffer, pH 7.6. Sections were slightly counterstained with Mayer’s hematoxylin.

### Immunohistochemical quantification

To estimate the intensity of ERβ immunostaining, computer-assisted image analysis was used based on previously reported protocols [33]. For each animal, four pictures from different areas of the ventral and dorsal prostate were obtained using a Nikon Eclipse E600 microscope (Nikon Corp, Melville, USA). Digital images were processed with Adobe Photoshop (Adobe Systems, Mountain View, USA), converted to the grayscale mode and inverted. The images were then exported to Image-J software (Image Processing and Analysis in Java, NIH Image, Bethesda, Maryland, USA) for quantitative analysis. For this experiment, 20 epithelial cell nuclei per area of atrophic, hyperplasic or PIN epithelium, as well as the adjacent normal epithelium, totaling approximately 480 nuclei for each animal, were traced, and the cell area was measured. The pixel intensity was determined for the traced areas. For each alveolus, one round nucleus was randomly chosen to be measured. Then, the next nineteen consecutive nuclei were quantified, except those that were out of focus, which were not considered. Background intensity was determined by tracing an unlabeled area adjacent to the measured cells. The final pixel intensity was calculated by subtracting the values detected in labeled nuclei from the background.

To estimate the proliferation rate, four pictures were taken at 40x magnification from five different regions of the ventral prostate, totalizing twenty images capture per animal. The images were processed using Adobe Photoshop and ImageJ program. The positive and negative epithelial cells were recorded, and the results expressed as a percentage of immunopositive cells [14]. Quantification of the intraepithelial proliferation areas (PIN) was performed by using a grid (area = 0.06 mm²) coupled to the light microscope (Nikon Eclipse E600, Japan) at 10x magnification. The results were expressed as number of PIN/mm².

### Immunofluorescence

To correlate the profile of cell proliferation and the ERβ expression, immunofluorescence was also performed, aiming to colocalize ERβ and proliferation markers Ki67. The sections were prepared as for immunohistochemistry. After the antigenic recovery the ventral prostate were permeabilizated in TBS containing triton X-100 0.5% (v/v) and washed in TBS. Non-specific binding was blocked by incubation with 5% goat serum and TBS/BSA 1% for 60 min. The tissues were incubated with the primary antibodies mouse monoclonal anti-human ERβ (NCL-ERβ, Leica Biosystems, Wetzlar, Germany) diluted 1:25 plus rabbit polyclonal anti-human Ki67 (ab15580, Abcam, Cambridge, USA) diluted 1:100 in TBS/BSA 1%, at 4°C, overnight. After washing, the sections were incubated with the secondary antibodies goat anti-mouse CF555 conjugated (Sigma-Aldrich—Dorset, UK) diluted 1:50 plus goat anti-rabbit FITC conjugated (F0382, Sigma-Aldrich—Dorset, UK) diluted 1:100 in TBS/BSA 1% at room temperature for 60 min. The negative control was performed in the absence of both primary antibodies. Nuclei were identified with 4,6-diamidino-2-phenylindole staining (DAPI—D1306, Life Technology, Carlsbad, USA). The sections were examined using a C2 Eclipse Ti confocal microscope (Nikon) with filters suitable for selectively detecting the fluorescence of FITC (green), Cy3 (red) or DAPI (blue).

Considering that the quantification of proliferative cells was performed by using MCM7 as marker, but both ERβ and MCM7 primary antibodies were raised in mouse, thus hindering colocalization, a parallel assay was made in serial sections stained for ERβ and MCM7 aiming to confirm the dual immunofluorescence of proliferating cells and ERβ (data not shown).

### Western blotting

Frozen prostatic lobes (n = 5 per age) were macerated using dry ice, and the pulverized tissue (100 mg) was homogenized in 300 μL of 8 M urea buffer containing 20 mM Tris-HCl pH 7.5, 0.5 mM EDTA pH 8.0, and protease inhibitor cocktail (Sigma-Aldrich, Dorset, UK). The samples were sonicated (Sonics & Materials, USA) on crushed ice and centrifuged for 10 min at 14000 g at 4°C for total protein extraction. Protein content was determined by the Bradford reagent using bovine serum albumin as a standard. Total protein extract (50 μg) was mixed with sample buffer containing 1% sodium dodecyl sulfate, 30 mM Tris-HCl pH 6.8, 2-mercaptoethanol, 20% (v/v) glycerol and bromophenol blue. After 5 min of boiling, the samples were subjected to continuous electrophoresis using 10% SDS-PAGE. A prestained protein molecular weight standard (PageRuler, Thermo Scientific, Waltham, USA) was used as a reference, and the separated proteins were transferred to a nitrocellulose membrane (Immobilon NC, Merck Millipore, Darmstadt, Germany). The membranes were blocked with 10% normal goat serum for 1 h at room temperature, incubated with a mouse anti-human ERβ antibody (NCL-ERβ, Leica Biosystems, Wetzlar, Germany) diluted 1:500 in PBS and incubated in a cold chamber overnight. After washing with PBS-0.05% Tween (PBST), the blots were incubated with a biotinylated goat anti-mouse antibody (Dako, Glostrup, DK) diluted 1:1000, followed by incubation with avidin-biotin conjugated to peroxidase (Vectastain Standard ABC kit, Vector Laboratories, Burlingame, USA). After several washes, the reaction was developed by the addition of 0.1% 3,3’diaminobenzidine in PBS containing 0.05% chloronaphthol, 16.6% methanol and 0.04% H_2_O_2_. The reaction was stopped with deionized water. The β-actin signal was used as the internal control. Band intensities were estimated using Image J software (NCBI, Bethesda, USA). Each prostatic lobe was measured in duplicate and repeated in two to three independent assays.

### Reverse transcription-PCR

Total RNA from the prostatic ventral and dorsal lobes (n = 5 per age) was extracted using Trizol reagent (Invitrogen, San Diego, USA), according to the manufacturer’s instruction. The samples were subjected to RT-PCR amplification using the Thermoscript RT-PCR kit (Invitrogen, San Diego, USA) for first strand cDNA synthesis. Oligo(dT)-primed cDNAs were synthesized from total RNA (2.5 μg) for 1 h at 55°C. The resulting cDNAs (1 μL) were amplified using PCR in a final volume of 10 μL containing 20 mM Tris-HCl (pH 8.4), 50 mM KCl, 1.5 mM MgCl_2_, 0.4 mM dNTPs, 2 units of Taq polymerase (Invitrogen, San Diego, USA) and 0.4 μM of each sense and antisense primer to amplify specific nucleotide sequences present in the ESR2 (ERβ) and *Gapdh* transcripts. The primer sequences for ERβ were as follows: forward primer, 5’ CTC ACG TCA GGC ACA TCA GT 3’; reverse primer, 5’ TGT GAG CAT TCA GCA TCT CC 3’ (NCBA reference sequence: NM_012754.1). The primer sequences for GAPDH were as follows: forward primer, 5’ AGA CAG CCG CAT CTT CTT GT 3’; reverse primer, 5’ CTT GCC GTA GGT AGA GTC AT 3’ (NCBA reference sequence: NM_017008).

Semi-quantitative RT-PCR analysis was used to determine the expression levels of each specific gene target. For each pair of primers, the number of cycles to amplify each cDNA in the linear range was determined under the following PCR conditions: an initial cycle of 3 min at 95°C, followed by 21–30 cycles of 1 min at 95°C, 1 min at 60°C, 1.5 min at 72°C, and a final extension of 3 min at 72°C.

DNA samples (9 μL) were loaded onto agarose gels (1.5% w/v) containing ethidium bromide (10 μg/mL). Densitometric analysis of RT-PCR bands was performed using Scion Image Analysis software (Scion Corporation—Frederick, USA). The results were calculated as the ratio of the signal of each specific gene in each sample to its corresponding internal control (*Gapdh*). The normalized data were then expressed as a percentage of the respective control group (mean ± SEM). To ensure reliability, PCR analysis for each gene was independently performed in duplicate for each tissue sample.

### Enzyme linked immune sorbent assay—ELISA

The dosage of 17β-estradiol and dihydrotestosterone (DHT) in the plasma and ventral prostate (n = 3 per age) was measured using commercial ELISA kits (DRG Instruments, Marburgo, Germany). Plasma samples were obtained after centrifugation of total blood (2200 g for 10 min) in heparin-coated tubes. ELISA measurements were performed using 25 and 50 μL of sample per well for estradiol and DHT, respectively, according to the manufacturer’s instructions. The assays were preceded by steroid enrichment based on lipid extraction using diethyl ether [34]. For the DHT assay, the tissue samples were diluted (1:4) in a volume of 200 μL of PBS. The sensitivities of the assay for 17β-estradiol and DHT were 9.7 and 6.0 pg/mL, respectively. All samples were measured in quadruplicate and repeated in two to three independent assays.

### Statistical Analysis

Body weight, prostate relative weights, nuclear ERβ staining intensity and hormonal measurements were statistically analyzed to detect possible differences between groups. Normal distribution of the data was tested. Statistical analysis was performed by multiple variance analysis using one-way ANOVA and a post-hoc Tukey’s test to compare more than two populations or Student's *t*-test to compare the means between two populations. Nonparametric data were analyzed using the Mann-Whitney and Kruskal-Wallis and Dunn’s post-hoc tests for comparisons between two or more populations, respectively. The data were expressed as the mean ± SEM, and differences were considered significant at p ≤ 0.05.

## Results

### Body and prostate lobe weights

The rat body weight increased from three to six months of age and stabilized from this age onward (Table 1). No age-related differences were detected in the relative weight of any fresh prostatic lobes or in the fixed dorsal and lateral prostates (Table 1). Conversely, the ventral prostates showed a progressive increase in their relative weights from three to 12 months of age and decreased significantly at 18 months when fixed organs were analyzed (Table 1). Similarly, a significant increase in the fixed lateral prostate was observed at six and 24 months of age.

**Table 1:** Body and relative weight of the prostatic lobes from fresh and fixed tissues. Results are the mean ± SEM. BW = body weights; VP = ventral prostate; DP = dorsal prostate; LP = lateral prostate; AP = anterior prostate.

	Fresh tissue (n = 5)					Fixed tissue (n = 5)				
Age (months)	3	6	12	18	24	3	6	12	18	24
**BW (grams)**	350.06 ± 6.62	*555.8 ± 14.11	*612.0 ± 13.29	*516.2 ± 26.84	*584.0 ± 21.45	367.0 ± 5.83	*512.8 ± 19.44	*561.6 ± 20.03	*520.2 ± 18.14	*525.1 ± 19.06
**VP (grams)**	0.162 ± 0.028	0.142 ± 0.006	0.161 ± 0.13	0.168 ± 0.025	0.119 ± 0.011	0.170 ± 0.006	0.198 ± 0.009	*0.216 ± 0.019	**0.144 ± 0.007	0.185 ± 0.006
**DP (grams)**	0.043 ± 0.003	0.035 ± 0.003	0.036 ± 0.004	0.045 ± 0.004	0.035 ± 0.005	0.066 ± 0.004	0.065 ± 0.004	0.067 ± 0.006	0.051 ± 0.005	0.069 ± 0.008
**LP (grams)**	0.034 ± 0.002	0.040 ± 0.006	0.037 ± 0.003	0.041 ± 0.006	0.031 ± 0.003	0.044 ± 0.002	*0.073 ± 0.008	0.057 ± 0.002	0.053 ± 0.006	*0.063 ± 0.003
**AP (grams)**	0.062 ± 0.005	0.067 ± 0.006	0.044 ± 0.008	0.064 ± 0.007	0.057 ± 0.008	0.074 ± 0.004	0.088 ± 0.006	0.085 ± 0.008	0.070 ± 0.003	0.071 ± 0.006

* = p ≤ 0.05 compared to 3 months and

** = p ≤ 0.05 compared to 6 months.

### Histopathology of the prostatic complex

**Fig 1:**
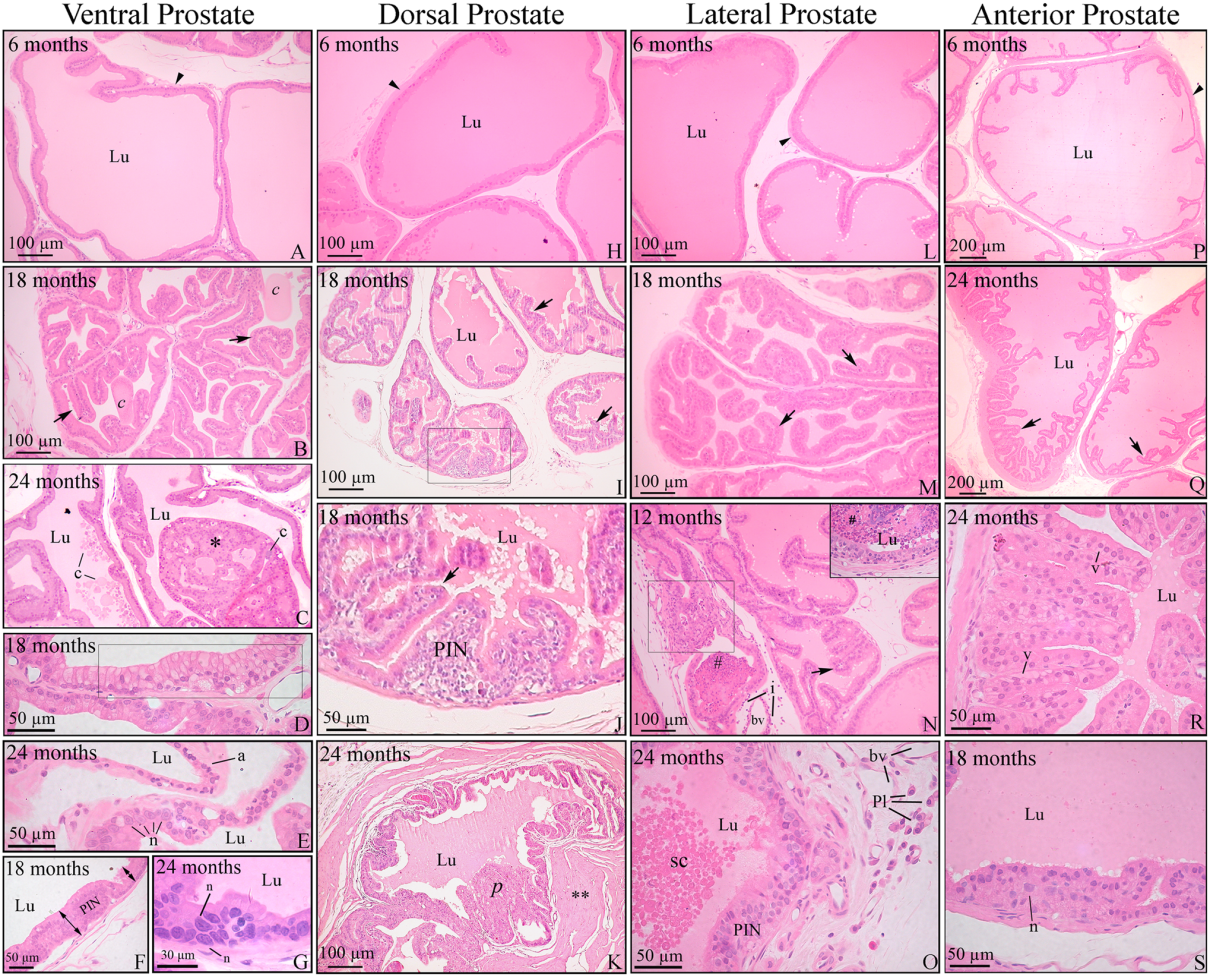
Histopathology of the prostatic complex of rats at different ages. Ventral (A-G); dorsal (H-K); lateral (L-O) and anterior (P-S) prostate. (A, H, L and P) Prostates of young adult rats showed normal histology. (B, I, M and Q) Senile rats showed increased unfolding of the epithelium (arrows) in all prostatic lobes. (B) Luminal concretions (c). (C) Epithelial proliferation resulted in cribriform architecture with intraluminal growing (*) in the ventral prostate. (D) Mucinous metaplasia of the epithelium (bounded area). (E) Epithelial atrophy (a) and area of a cell with nuclear enlargement and prominence of nucleoli (n). (F and G) Hyperproliferative epithelium with characteristic foci of prostatic intraepithelial neoplasia (PIN). (G) Cells with evident nuclear enlargement (n) in the PIN area. (J) Detail of the prostatic intraepithelial proliferation observed in the bounded area of the dorsal prostate in panel I. (K) Epithelial proliferation with papillary growth. Thickening of the stroma (**) occurred around the lesion. (N) Lateral prostate with epithelial hyperplasia (arrow) as well as foci of inflammatory cells infiltrating the epithelium (bounded area), lumen (#) and stroma (i). (O) PIN area next to an inflammatory cell infiltrate containing plasma cells (Pl). Cells sloughed into the lumen (sc). (R) Intense unfolding and vacuolization in the epithelium of the anterior prostate (v). (S) PIN area in the anterior prostate with some nuclear atypia (n). Arrowhead = perialveolar smooth muscle cell; Pl = plasma cells; bv = blood vessel; c = luminal concretions; Lu = lumen. Stained with H&E.

Compared to adult animals at three to six month of age, morphological alterations were observed in all prostatic lobes from 12 to 24 months (Fig 1). The changes were heterogeneous among animals of the same age and even within the glands of the same animal, in which normal acini colocalized with altered ones. The initial changes included slight to moderate unfolding of the acinar epithelium, which is suggestive of hyperplasia. Intraluminal concretions and cellular atypia, such as cytoplasmic vacuolization, as well as nuclear enlargement and the prominence of nucleoli were also observed (Fig 1). From 18 to 24 months of age, the prostatic concretions and the areas of hyperplasia and cellular atypia were more frequently observed and pronounced than those observed at 12 months of age (Fig 1B, 1I, 1M and 1Q), except for the anterior prostate in which the concretion contents did not vary with age. Proliferation of the epithelium led to cribriform or papillary architecture in some acini, especially in the ventral prostate (Fig 1C).

In addition to these common changes, the ventral prostate also presented areas of epithelial atrophy (Fig 1E). The atrophic acini were mostly dilated and lined with cubic to squamous epithelium. Areas of gradual changes from normal to squamous epithelium were frequently observed. In addition to atrophy, some cells of the ventral and dorsal prostate were characterized by cytoplasmic filling with mucinous material. These mucinous cells occurred scattered throughout the epithelium or in small group formations, indicative of mucinous metaplasia, as observed in the ventral prostate (Fig 1D).

**Fig 2:**
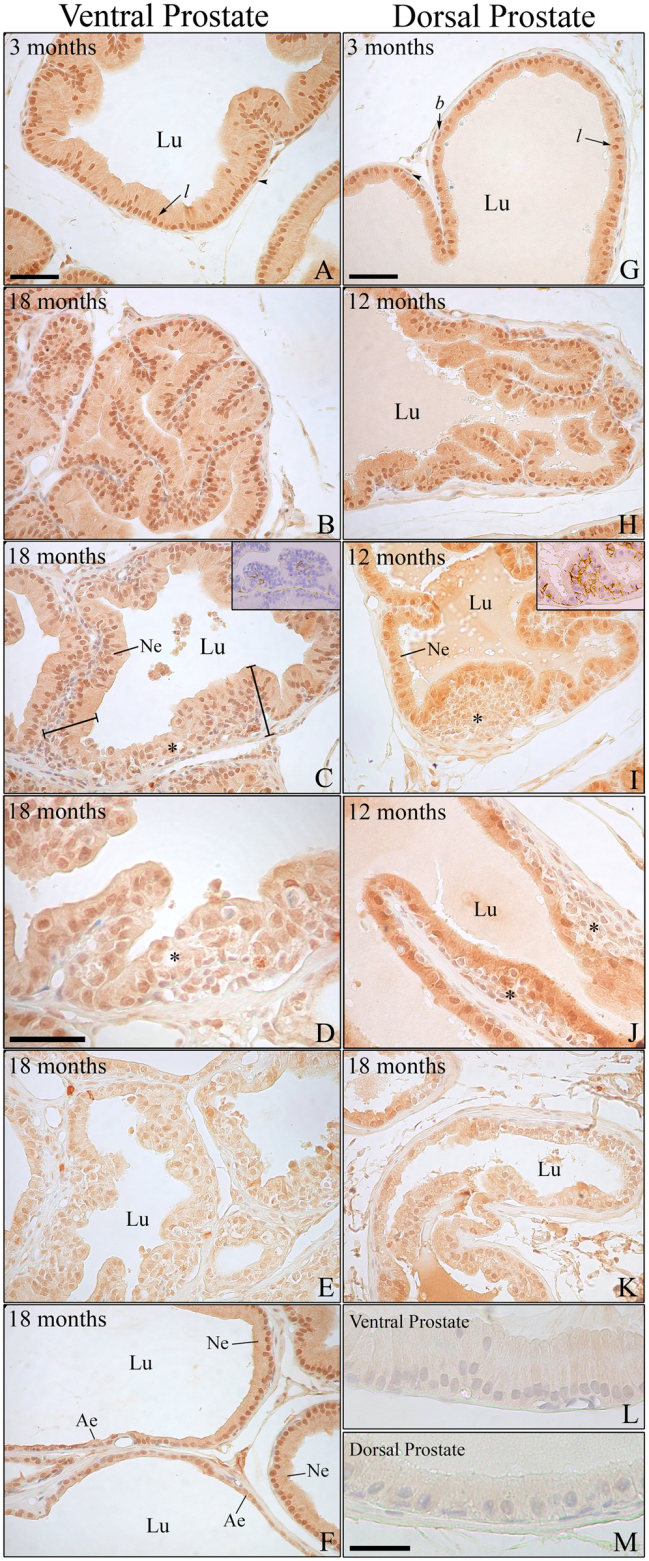
Immunostaining for ERβ is affected in specific areas of alterations related to aging. (A–F) Immunostaining for ERβ in the ventral and (G–K) dorsal prostate of rats at different ages. (A and G) Young adult animals showing intense positivity for the receptor in the epithelial luminal (*l*) and basal cells (*b*). Black arrowhead = positive perialveolar smooth muscle cells. (B and H) Senile rats presenting unfolding of the epithelium with normal ERβ expression. (C–D and I–J) Areas of intraepithelial proliferation (*) in the ventral and dorsal prostate, respectively, showing reduced ERβ staining compared with the normal epithelium (Ne). Insert in C and I: immunostaining for CK HMW in the ventral (C) and dorsal (I) prostate showing that the dorsal prostate presents a greater number of basal cells in intraepithelial proliferating areas. (E and K) Drastic reduction of ERβ immunostaining in areas of intense cellular atypia. (F) Transition from normal epithelium (Ne) showing standard ERβ positivity to atrophic epithelium (Ae) presenting a marked decrease in receptor staining. (L and M) Negative immunostaining controls for the ventral and dorsal prostate, respectively. Lu: lumen. Bar in A and G (= B, C, E, F, H, I, J, K) = 50 μm; bar in D = 40 μm; bar in M (= L) = 30 μm.

In the ventral, dorsal and lateral prostate, areas of intraepithelial stratification occurred, with preserved basement membrane and often with atypical cells, which were compatible with prostate intraepithelial neoplasias (PIN) (Fig 1F, 1G, 1J, 1O, 1S and insert in 1N). In the dorsal lobe, the proliferation was primarily in the basal compartment (Fig 1J). The identity of these cells as basal cells was confirmed according to CK HMW positivity (insert in Fig 2I).

In the anterior prostate, the areas of PIN were less frequently detected. Furthermore, in the lateral prostate, there were frequent inflammatory foci in the stroma as well as invading the acinar epithelium and lumen in the older rats, which is indicative of prostatitis (Fig 1N and 1O). Prostatitis in other lobes was infrequent.

### Immunolocalization of ERβ in the prostatic complex

Intense ERβ immunoreactivity was detected in the nuclei of luminal and basal epithelial cells of the ventral, dorsal (Fig 2) and anterior prostate (data not shown). Moderate immunoreactivity in the cytoplasm was also detected. The perialveolar smooth muscle cells presented intermittent expression of the receptor, whereas some stromal and endothelial cells were also positive for ERβ. Differing from other lobes, the lateral prostate presented a very intense cytoplasmic reaction that covered the nuclei. Several tests for blocking the unspecific reaction were tried without success. Therefore, data from this lobe could not be collected.

**Fig 3:**
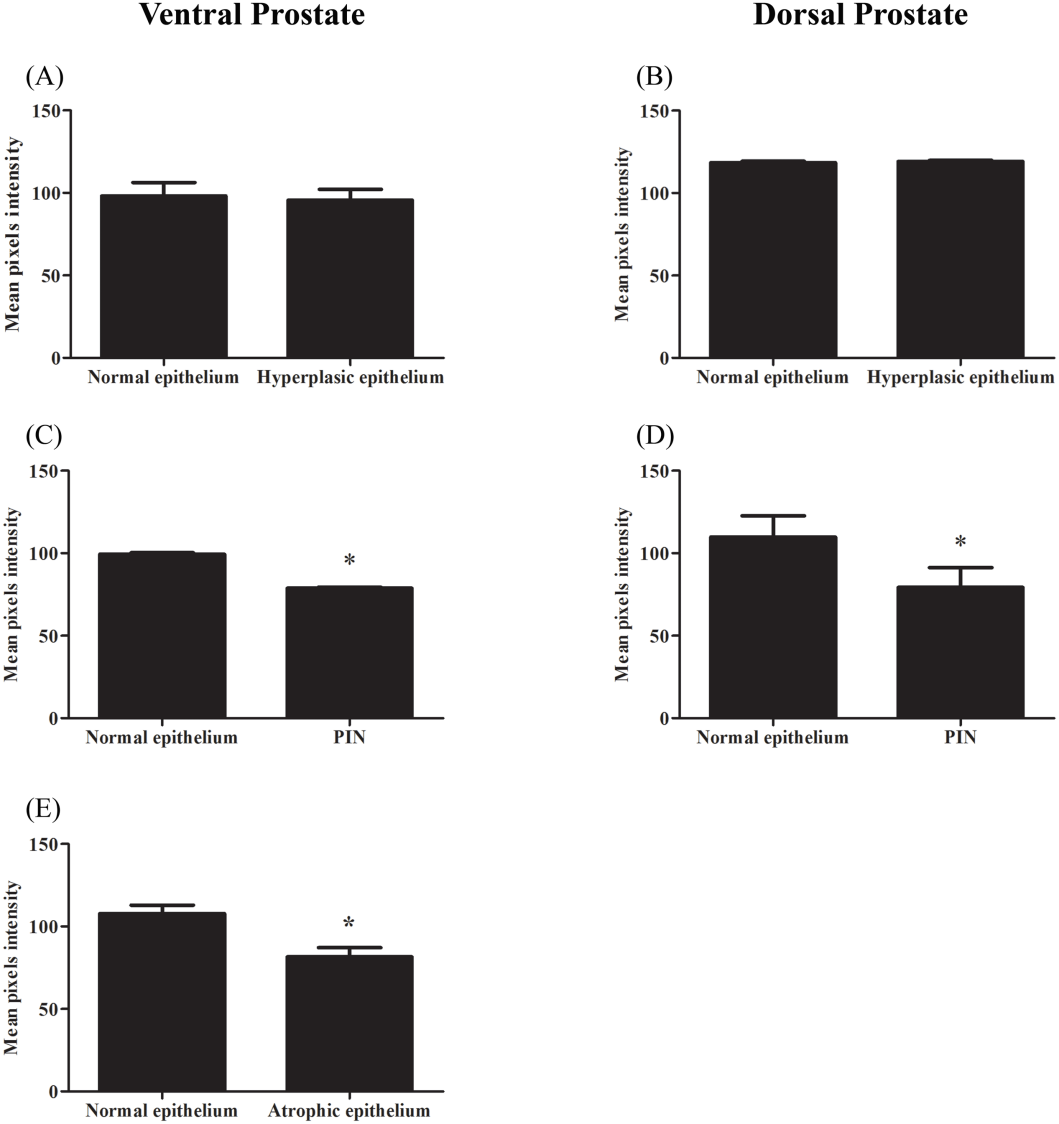
Quantification of ERβ immunoreactivity in the ventral and dorsal prostates of rats at different ages. (A–B) Compared to the normal adjacent epithelium, the areas of hyperplasia were similarly stained for ERβ, whereas those of PIN (C–D) and atrophy (E) showed decreased ERβ immunoreactivity. * = p ≤ 0.05; n = 4 per group.

The pattern and intensity of ERβ immunoreactivity did not change with age, when considering the epithelium of acini with normal morphology or those with hyperplasia (Figs 2B and 2H and 3A and 3B). On the other hand, the ERβ reaction was significantly reduced in areas of intraepithelial neoplasia, especially in those PIN where cellular atypies were predominant (Fig 3C and 3D). In these focal areas, a heterogeneous pattern of staining as weakly stained or unstained cells was observed along with cells positive for the receptor (Fig 2C, 2D, 2I and 2J). In the ventral prostate, the atrophic epithelium was also weakly stained (Figs 2F and 3E). Accordingly, in areas of change from normal to squamous epithelium, ERβ staining intensity gradually reduced (Fig 2F).

### Western blotting

**Fig 4:**
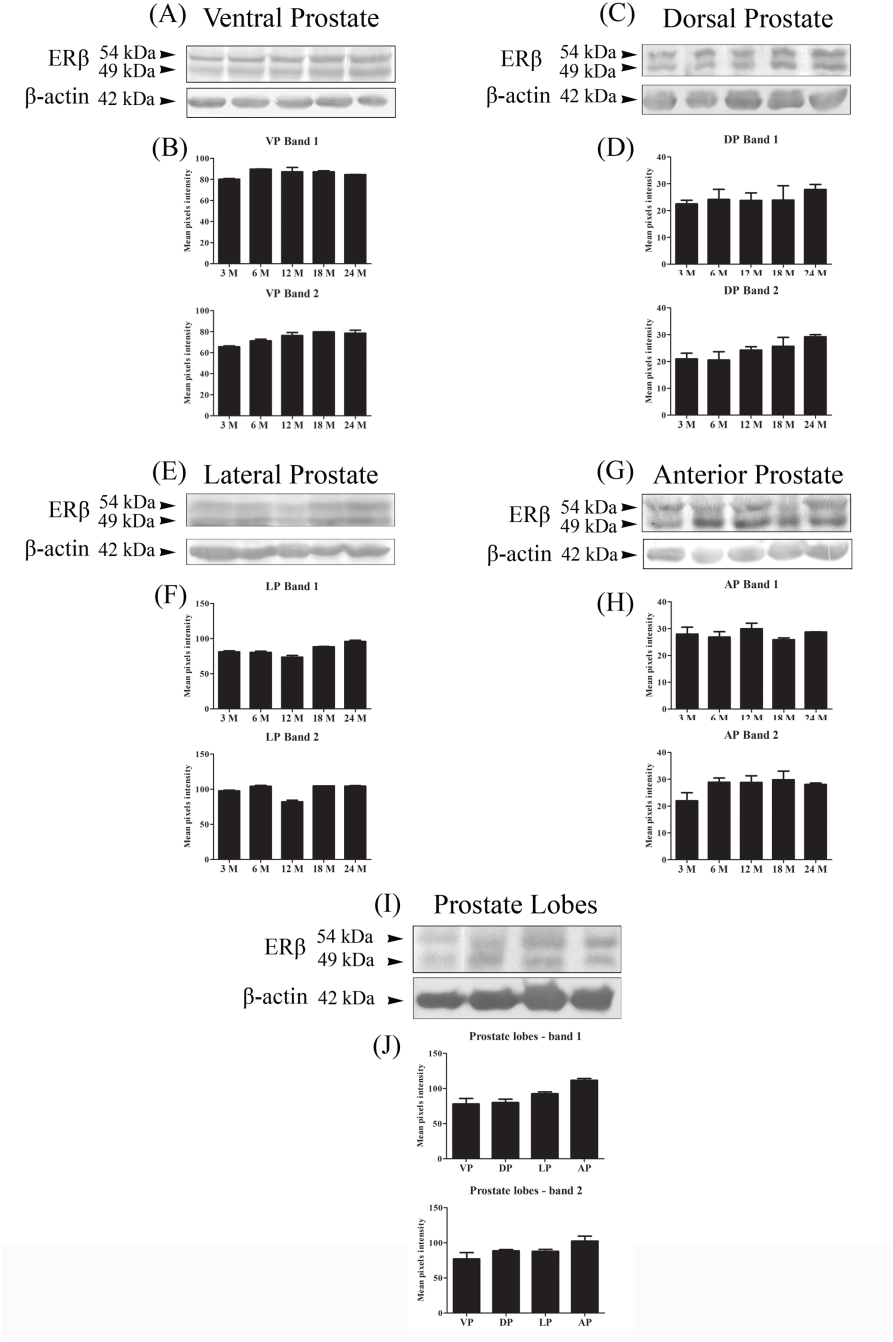
Western blotting of ERβ in the rat prostates at different ages. (A, C, E and G) Representative bands from the assay. β-actin was used as a reference. (B, D, F and H) Graphical representation of the band densitometric analysis. The data shown are representative of two to three different assays. VP = ventral prostate; DP = dorsal prostate; LP = lateral prostate and AP = anterior prostate. n = 5 per group.

The specificity of the antibody was confirmed according to the detection of a principal band of 54 kDa within the expected molecular mass previously described for rat ERβ [3, 35]. A second reactive band of 49 kDa, however, co-migrated with the principal band (Fig 4). The pixel intensity of the ERβ bands was similar among adult and senile rats (Fig 4A–4H) as well as among the different lobes (Fig 4I and 4J).

### Reverse transcription-PCR

**Fig 5:**
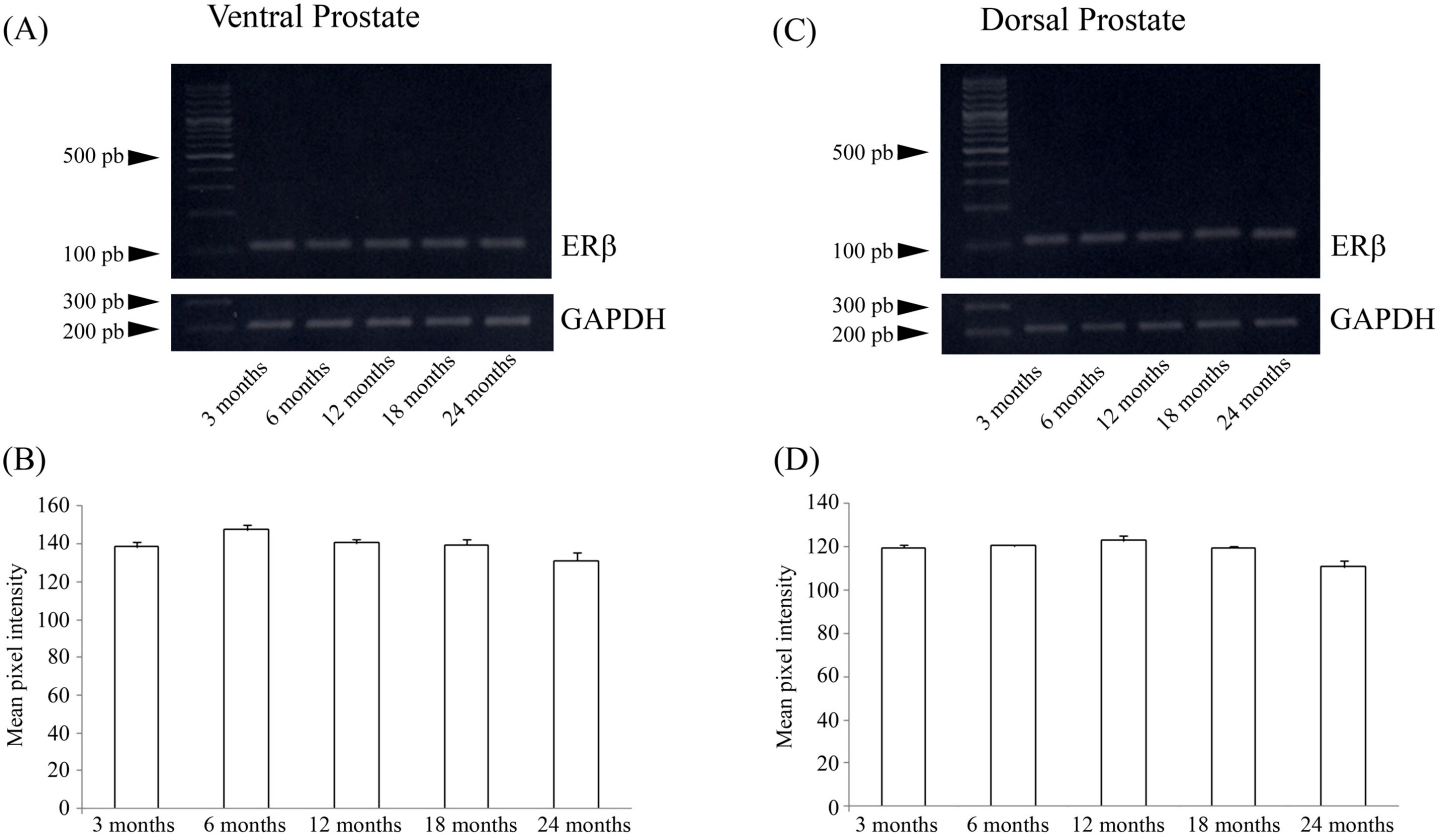
ERβ mRNA levels in the rat ventral and dorsal prostates at different ages. (A and C) Representative bands from the assay. Gapdh was used as a reference. (B and D) Graphical representation of the band densitometric analysis. The data shown are representative of two different assays. n = 5 per group.

No detectable alterations in the mRNA levels of ERβ was observed in the ventral and dorsal prostate (Fig 5).

### Cell proliferation

**Fig 6:**
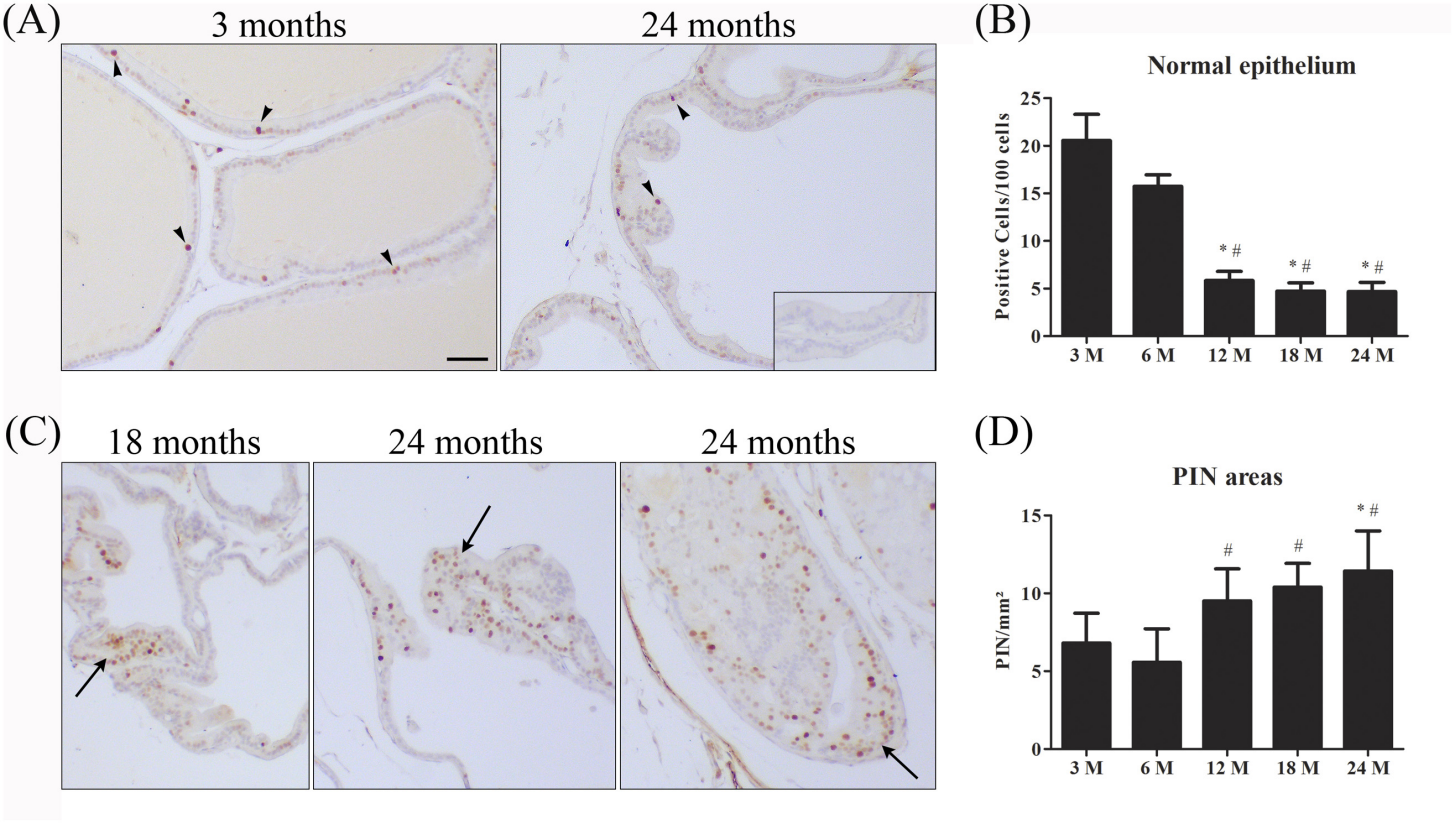
Immunostaining and quantification of MCM7 positive cells in the rat ventral prostate at different ages. (A) Immunopositivity for MCM7 in normal epithelium of young and senile rats. (B) Graphical representation of quantification of MCM7-positive cells in normal epithelium showing reduced number of positive epithelial cells as rat age. (C) MCM7 immunopositivity in PIN areas of aging prostates. (D) Graphical representation of MCM7-positive cells showing an increase of positive cells in PIN areas (arrows). * and # = p ≤ 0.05 compared to 3 and 6 months, respectively; n = 5 per group. Arrowhead = MCM7 positive cells. Insert: negative control. Bar = 50μm.

Considering the putative antiproliferative activity of ERβ, the profile of cell proliferation was evaluated owing to get a better insight into the possible biological significance of focal ERβ reduction in the aging prostates. Few MCM7 positive cells were found in the normal epithelium of the ventral prostate of animals from 3 to 24 month of age. When compared to the young animals (3–6 month), as rats age, there was a gradual reduction in the number of proliferative cells in the normal epithelium (Fig 6). However, from 12 month onward a growing number of adenomers presented higher frequency of MCM7 positive cells, which were restricted to areas of intraepithelial proliferation (PIN) (Fig 6).

**Fig 7:**
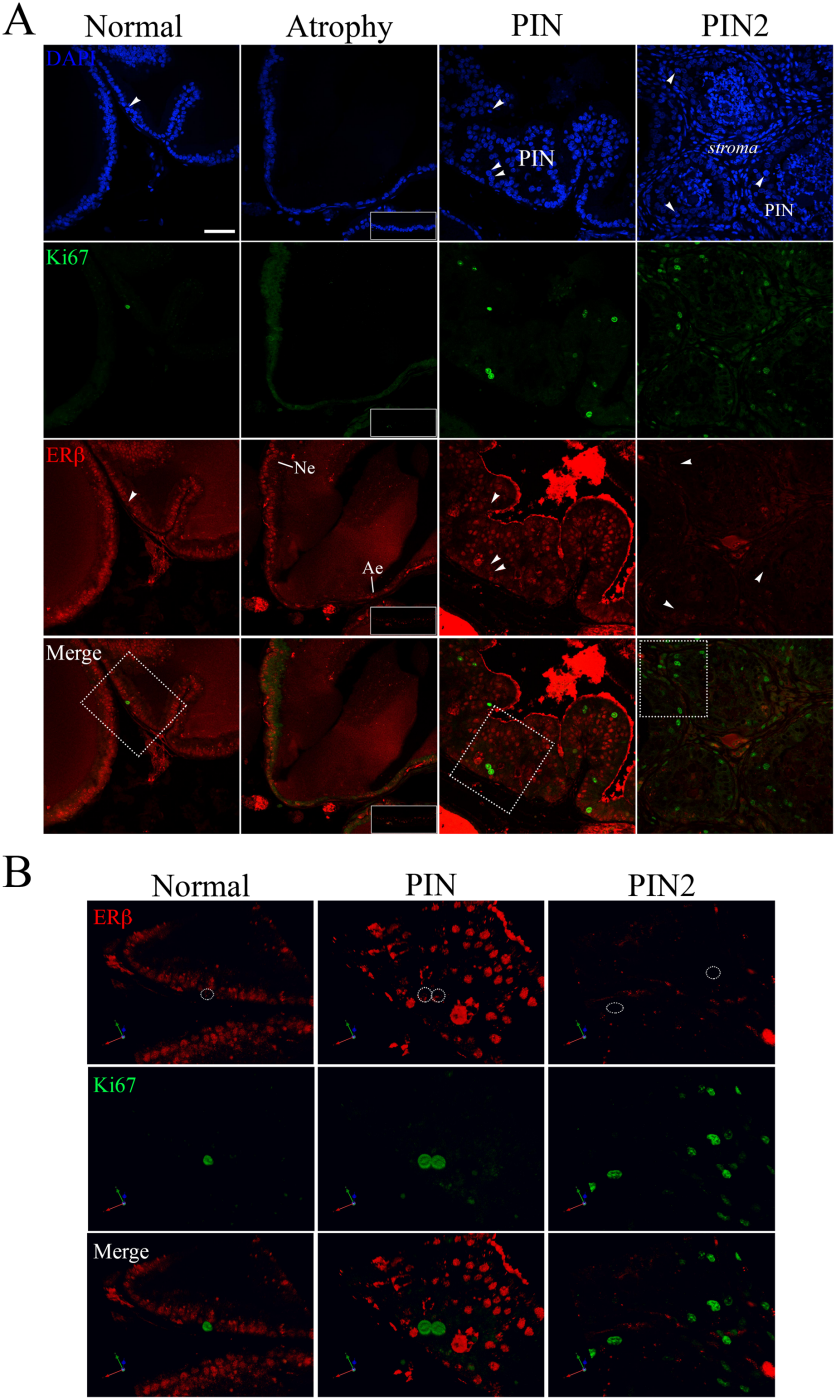
Colocalization of ERβ and the proliferation marker Ki67 in the ventral prostate of senile rats. (A) Comparison between normal epithelium (Ne), atrophic epithelium (Ae) and PIN areas. In PIN areas the ERβ immunoreaction is reduced especially in cells with nuclear atypies (PIN2), whereas the proliferating cells (arrowhead) were more frequently found. (B) 3D reconstruction of the areas demarcated in A. Inserts correspond to negative controls of the respective channels. Bar = 50 μm.

The fluorescence data corroborated the immunohistochemistry results. Few Ki67 positive cells were detected in normal epithelium, whereas in PIN areas the Ki67 positive cells were more frequently found. The ERβ fluorescence was constant in normal epithelium and reduced in atrophic epithelium (Fig 7A). In the PIN areas the ERβ fluorescence was heterogeneous with weakly reactive or unreactive nuclei found in the same area. The colocalization of ERβ and Ki67 revealed that the proliferating cells have low to undetectable positivity for ERβ, corroborating the antiproliferative profile of this receptor. Interestingly, in the PIN areas where nuclear atypies were predominant, most cells were unreactive for ERβ whereas the Ki67 positive cells were frequent (Fig 7B).

### Hormone levels

**Fig 8:**
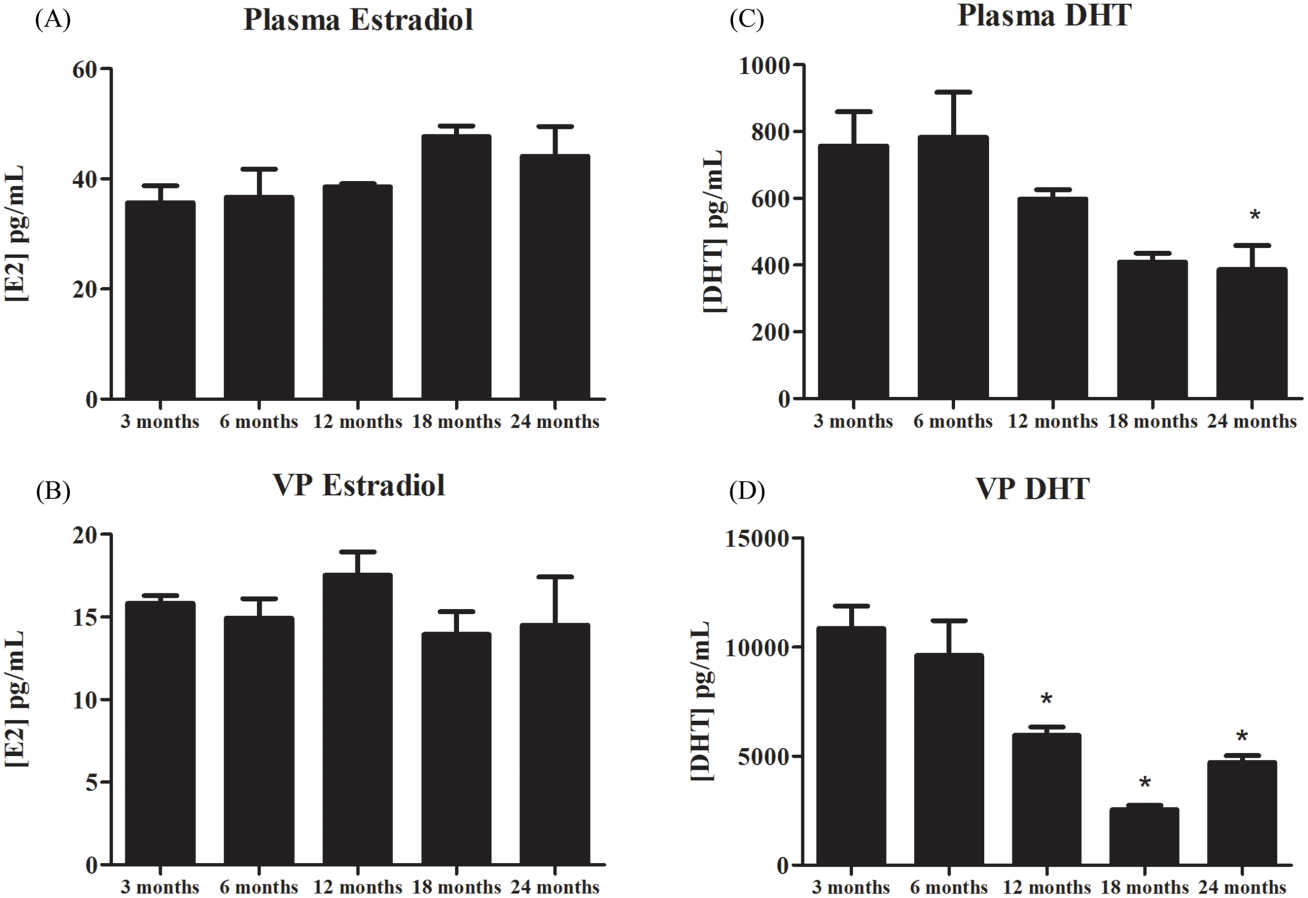
Plasmatic and tissue hormonal levels of 17β-estradiol (E2) and dihydrotestosterone (DHT) of rats at different ages. (A and B) Levels of E2 in the plasma and ventral prostate. (C and D) Levels of DHT in the plasma and ventral prostate. The data shown are representative of two to three different assays. VP = ventral prostate. * = p ≤ 0.05; n = 3 per group.

The concentration of estradiol in the plasma and ventral prostate tissue was similar in rats from 3 to 24 months of age (Fig 8A and 8B). Conversely, the DHT levels were gradually reduced with age in both the plasma and ventral prostate (Fig 8C and 8D).

## Discussion

Remarkable morphological alterations were observed in Wistar rat prostate from 12 months of age onward, and these changes included hyperplasia, foci of intraepithelial proliferation, and nuclear atypies of epithelial cells in all lobes, as well as epithelial atrophy in the ventral prostate and inflammation especially in the lateral prostate. A reduction in ERβ staining was observed in specific areas and was related to intraepithelial proliferation, cellular atypies and atrophic abnormalities. The present results add to findings regarding focal ERβ reduction in pre-malignant/malignant disorders in human and dog prostates, suggesting the association of prostatic pathologies with a putative disorder in the ERβ pathway.

Alterations in the structure of prostatic glands were observed in every component of the aging prostatic complex of Wistar rats. Intraluminal concretions, epithelial hyperplasia, nuclear atypia and intraepithelial neoplasia were common morphological changes among the prostate lobes. The ventral prostate was the most affected lobe, which in addition to the above changes, was also characterized by frequent epithelial atrophy that was concomitant with luminal dilation, and characterizes cystic acini [32]. Conversely, the anterior prostate was the least affected lobe. These spontaneous alterations corroborate previous data concerning the aging prostate in several lineages of rats and other rodents, such as Brown Norway rats [28], Lobund/Wistar rats [36], ACI/Seg rats [37, 38], Fisher 344 rats and B6C3F1 mice [39], Noble rats [40,41], gerbils [42] and C57BL/6 mice [43]. Together, these data note that the aging rodent model may be a natural and effective tool to study disorders related to the prostate.

Intense ERβ immunoreactivity was detected in the nuclei of basal and luminal epithelial cells as previously described [4–5, 33]. No difference in staining intensity was detected when areas of normal epithelium or hyperplasia were considered. However, a significant reduction in ERβ immunostaining was found in focal areas of cellular atypia and intraepithelial proliferation, especially in the ventral and dorsal prostate. A similar reduction in ERβ was detected in the atrophic epithelium of the ventral prostate. A reduction in ERβ expression has also been described in premalignant and malignant lesions of human and dog prostates [6, 16–17, 19–23, 44–46]. In humans and dogs, the intensity of prostate ERβ expression is related to the degree of tumor differentiation as less differentiated tumors are more prone to a reduction in protein expression [16, 18–21, 46–47]. The lack of ERβ in βERKO mice also resulted in increased PIN lesions later in life [48]. PIN represents a precancerous lesion that can progress to latent cancer and ultimately to malignant cancer [48]. Although prostate cancer is a disease common in elderly men, PIN and microscopic cancer foci can be detected as early as 25–30 years of age in males of all ethnic groups [48]. The similarity of the rat model may be important for future experimental investigations into possible precocious therapeutics for controlling later prostate cancer.

ERβ has been associated with the pro-apoptotic, anti-proliferative and pro-differentiation roles [8, 9, 10, 11, 12, 14, 15, 49, 50, 51]. Corroborating the involvement of the receptor with proliferative activity, we presently found that the punctual reduction in ERβ paralleled the increase in cell proliferation especially in areas of PIN and nuclear atypies. Similarly others have found low to undetectable levels of ERβ in proliferating cells of rodent ventral prostate [9]. These data are important considering that similar alterations have been described for human prostate, in which ERβ is highly decreased in PIN areas and adenocarcinomas, reaching undetectable levels with the tumor progression, even though the receptor prevail in hyperplasic tissue [6, 7, 16, 17, 18, 19, 44]. There are evidences that PIN and latent microfoci of cancer can be recognized in men as early as 20–30 years of age, even before the clinically relevant carcinogenic lesions to be detected commonly beyond the age of 50 [48,52]. On this sense, the present findings substanciate the importance of ERβ as potential target for management of proliferative disorder of the prostate at premalignant phases.

The decrease in ERβ in the rat prostate occurred in a hormonal milieu characterized by a steady concentration of estradiol and decreased plasmatic and tissue DHT. A reduction in androgen and increase or maintenance of the estradiol levels in the plasma and tissue has long been postulated as a risk factor for pathological changes in the prostate [28,53–54], thus corroborating our findings. The reduction in ERβ without changes in the local estradiol levels indicates that the receptor does not appear to be modulated by estradiol. Others have also found that estrogens do not autoregulate ERβ in the rat prostate [31, 33]. On the other hand, evidence suggests that estradiol is not the only estrogen acting in the prostate. In fact, the most abundant estrogenic steroid in this tissue is 5α-androstane, 3β,17β-diol (3β-diol), a DHT metabolite that acts by paracrine and autocrine mechanisms [55]. The decrease in DHT may have resulted in a decrease in 3β-diol, which has been shown to modulate the expression of ERβ in the rat prostate [33]. In contrast to 3β-diol, the cognate ligand of ERβ, estradiol, had minor effects on the ERβ levels [33]. In addition, ERβ autoregulation by 3β-diol has also been found in other organs [56]. Therefore, an investigation of the prostate 3β-diol levels during aging is warranted to clarify the composition of the hormonal milieu that leads to local alterations in this gland.

The Western blotting assays detected ERβ as a duplet at approximately 54 and 49 kDa in the rat prostate extracts. Similar results have been described for other species, such as humans [16], primates [57], mice [58], pigs [59] and turtles [60]. These two protein bands are suggested to represent the translation of ERβ from two initiation codons, named long and short ERβ [16, 57]. Long ERβ represents the protein translated from the first initiation codon of the transcript, whereas short ERβ results from the translation that starts from the second initiation codon [16]. These proteins are functionally similar [16, 61] and differ from ERβ splice variants, which usually have deletions or insertions of amino acids in their terminal region, resulting in proteins with distinct function [62–65]. Notably, the expression profile of both ERβ bands was similar in all lobes analyzed.

This research is a pioneering study that revealed focal changes in ERβ expression in the prostatic complex of aging rats. The reduction in ERβ in selected areas associated with intraepithelial neoplasia, cellular atypia and atrophy indicates a potential disorder in the ERβ pathway, corroborating previous data from humans and dogs that silencing of this receptor may be associated with premalignant or malignant conditions in the prostate. Additionally, the results underscore the importance of careful tissue-based analysis of estrogen receptor expression in the prostate to better understand the alterations related to prostatic diseases.
